# VEGF-Mediated Augmentation of Autophagic and Lysosomal Activity in Endothelial Cells Defends against Intracellular Streptococcus pyogenes

**DOI:** 10.1128/mbio.01233-22

**Published:** 2022-07-05

**Authors:** Shiou-Ling Lu, Hiroko Omori, Yi Zhou, Yee-Shin Lin, Ching-Chuan Liu, Jiunn-Jong Wu, Takeshi Noda

**Affiliations:** a Center for Frontier Oral Science, Graduate School of Dentistry, Osaka University, Osaka, Japan; b Research Institute for Microbial Disease, Osaka University, Osaka, Japan; c Department of Microbiology and Immunology, College of Medicine, National Cheng Kung University, Tainan, Taiwan; d Center of Infectious Disease and Signaling Research, College of Medicine, National Cheng Kung University, Tainan, Taiwan; e Department of Pediatrics, College of Medicine, National Cheng Kung University, Tainan, Taiwan; f Department of Biotechnology and Laboratory Science in Medicine, College of Biomedical Science and Engineering, National Yang Ming Chiao Tung University, Taipei, Taiwan; g Graduate School of Frontier Biosciences, Osaka University, Osaka, Japan; Ohio State University

**Keywords:** VEGF, TFEB, endothelial cells, *Streptococcus pyogenes*, group A *Streptococcus*

## Abstract

Group A Streptococcus (GAS), a deleterious human-pathogenic bacterium, causes life-threatening diseases such as sepsis and necrotic fasciitis. We recently reported that GAS survives and replicates within blood vessel endothelial cells because these cells are intrinsically defective in xenophagy. Because blood vessel endothelial cells are relatively germfree environments, specific stimulation may be required to sufficiently induce xenophagy. Here, we explored how vascular endothelial growth factor (VEGF) promoted xenophagy and lysosomal activity in endothelial cells. These effects were achieved by amplifying the activation of TFEB, a transcriptional factor crucial for lysosome/autophagy biogenesis, via cAMP-mediated calcium release. In a mouse model of local infection with GAS, the VEGF level was significantly elevated at the infection site. Interestingly, low serum VEGF levels were found in a mouse model of invasive bacteremia and in patients with severe GAS-induced sepsis. Moreover, the administration of VEGF improved the survival of GAS-infected mice. We propose a novel theory regarding GAS infection in endothelial cells, wherein VEGF concentrations in the systemic circulation play a critical role.

## INTRODUCTION

Streptococcus pyogenes, also known as group A Streptococcus (GAS), is a common human infectious pathogen that causes a wide spectrum of illnesses. These range from mild, localized diseases, such as pharyngitis and impetigo, to life-threatening invasive conditions that include sepsis, necrotic fasciitis, and streptococcal toxic shock syndrome (1, 2). During the past 2 decades, GAS-induced infectious diseases have become more invasive (2). In particular, the clinical progression in severe cases is often so rapid that antibiotics are ineffective. On the other hand, the development of vaccines against GAS remains challenging, mainly due to the existence of numerous serotypes of M protein, a type of GAS major surface protein, as well as ineffective immune responses against GAS particles or toxins in a mouse model (3). Therefore, the pathogenesis of GAS-induced invasive disease must be further clarified to facilitate the development of novel treatments.

Although GAS was initially identified as an extracellular bacterium, it was later also found to invade host cells to escape phagocytosis and antibiotic-induced death; even its efficacy was thought to be not high (4–7). Once GAS invades host cells via the host cellular endocytic pathway, it is able to escape from endosomes/lysosomes into the cytoplasm by creating membranous pores using hemolysins such as streptolysin O (SLO) or streptolysin S (SLS) (8, 9). Autophagy is a cellular homeostatic pathway that engulfs and degrades self-components and organelles (10, 11). In epithelial cells, escaped GAS is engulfed by autophagosomes, the membrane structures involved in the autophagy pathway, and is then killed in autolysosomes, which are organelles formed by the fusion of autophagosomes and lysosomes. Autophagy can suppress GAS proliferation in epithelial cells (8, 9), although several virulence factors secreted by GAS help defend against this process (12, 13).

Severe symptoms of invasive disease are closely associated with the breakdown of blood vessel barriers and the subsequent dissemination of bacteria into the systemic circulation. Damage to vessel barriers can be caused by virulence factors and/or GAS invasion into endothelial cells, and the resulting cell death. We previously reported that the invasive efficacy of GAS is 5-fold higher in endothelial cells than in epithelial cells (14, 15). Furthermore, blood vessel endothelial cells failed to suppress intracellular bacterial proliferation due to their limited efficacy in terms of autophagy and lysosomal function (14, 15). These cells did not demonstrate effective ubiquitination of GAS, which is necessary for subsequent autophagy progression (15). Furthermore, they exhibited attenuated lysosomal acidification, which led to high hemolysin SLO expression and GAS proliferation (14).

Thus, there exists a clear distinction between endothelial cells and epithelial cells regarding their capacity to neutralize bacteria. Understanding this aspect of endothelial cells should facilitate the development of preventive treatments for severe GAS infection. In the present study, we reveal that vascular endothelial growth factor (VEGF) plays a pivotal role in GAS clearance in endothelial cells. Among various VEGF subtypes, VEGFA165 is the most ubiquitously expressed in most tissues, and is, therefore, in this report referred to simply as VEGF (16). It is involved in stimulating endothelial cell survival and proliferation, and is required for angiogenesis, vascular permeability, inflammation (17), and even aging (18). VEGF is also related to tumorigenesis, metastasis, and blinding eye disease (19, 20). Activation of VEGFR-1/2, a tyrosine kinase cell receptor heterodimer expressed primarily on endothelial cells, leads to an intracellular signaling cascade that is strongly related to the biological functions of endothelial cells (21). In this study, we explored how VEGF affects endothelial cells in the context of GAS infection, and further examined its pathophysiological role *in vivo*.

## RESULTS

### VEGF enhanced GAS clearance in endothelial cells.

**FIG 1:**
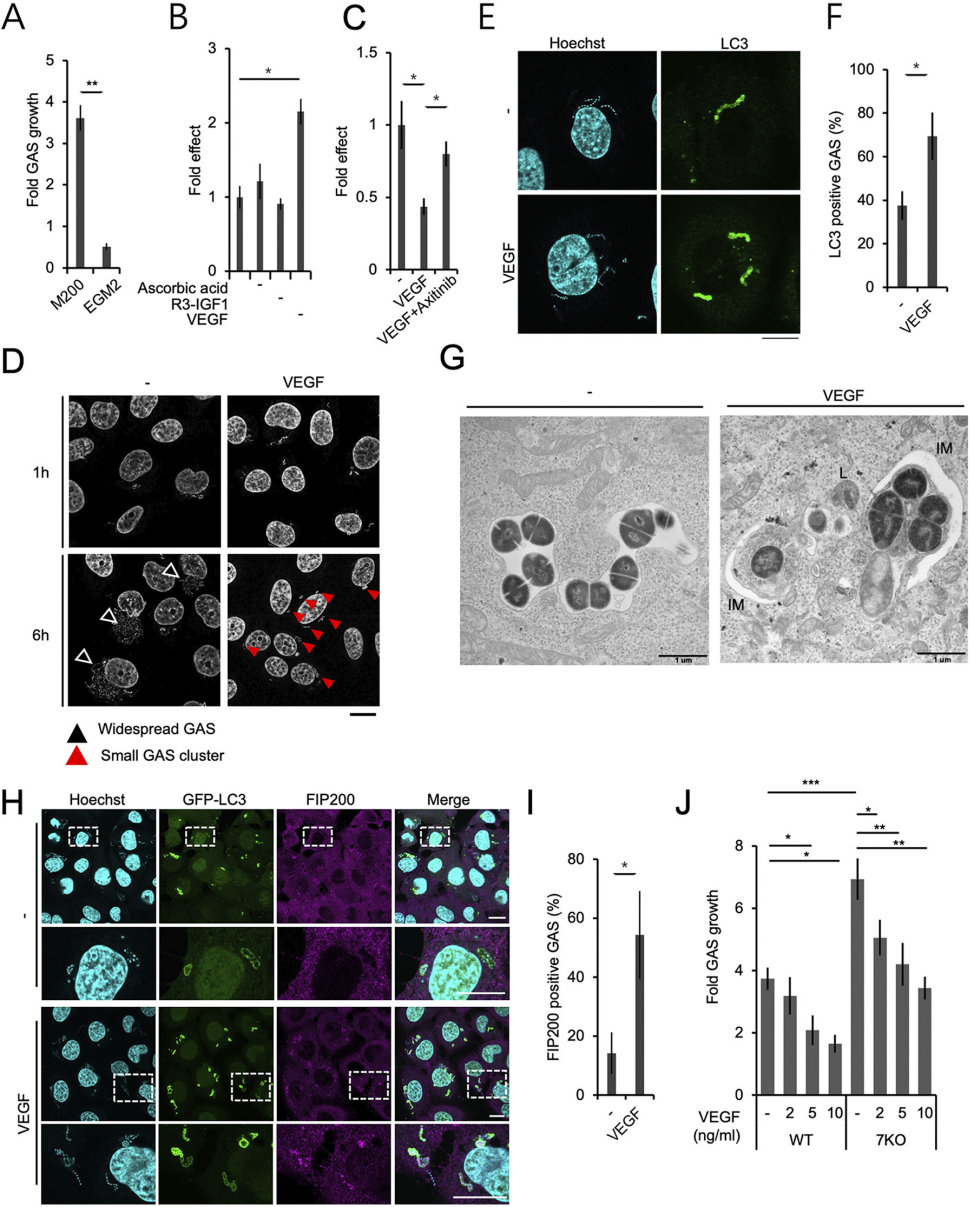
VEGF treatment ameliorated GAS clearance in endothelial cells partly by promoting xenophagy. Intracellular growth/survival of GAS in endothelial HMEC-1 cells was determined under different cell culture conditions. (A) Endothelial cells were maintained in M200 or EGM2 for 2 d and then infected with GAS. The ratio of the intracellular GAS count at 6-h/1-h postinfection was determined by colony forming assay (CFA). (B) HMEC-1 cells were maintained in EGM2 or in versions of EGM2 medium lacking the indicated ingredients, and were infected with GAS to perform CFAs as above. (C) HMEC-1 cells were maintained in M200 with or without 1 nM VEGF and/or 10 μM axitinib, and infected with GAS to perform CFAs as above. (D) GAS-infected HMEC-1 cells were fixed at the indicated time points postinfection and stained with Hoechst for confocal microscope observation. Antibiotic was continually present in the culture medium after 30-min GAS inoculation. Black arrowheads represent widespread GAS; red arrowheads represent clustered GAS. (E–G) HMEC-1 cells cultured in M200 and infected with GAS for 1 h were stained with anti-LC3 antibodies (E and F) for confocal microscopy observation or prepared for transmission electron microscopy observation (G) (IM, isolation membrane; L, lysosome). (H) HMEC-1 cells expressing GFP-LC3 were infected with GAS for 1 h and stained with anti-FIP200 antibody. The graph represents the percentage of LC3- or FIP200-positive GAS per total intracellular GAS (F and I). (J) Wild-type and ATG7 KO HMEC-1 cells maintained in M200 with or without VEGF at the indicated concentrations. Fold increase in GAS growth was calculated by CFA as the colony number at 6 h normalized to that at 1-h postinfection. Fold of effect was further normalized to the control group (B and C). Scale bars are 10 μm for fluorescent images and 1 μm for EM images. All quantitative data represent the mean and SD from three independent experiments. *, *P < *0.05; **, *P < *0.01; ***, *P < *0.001 by unpaired two-tailed Student’s *t* test.

Endothelial cells require specific nutrients and growth factors for their survival and proliferation, and thus specially optimized culture media are commercially available from various suppliers. In previous studies, we utilized M200 medium with 10% serum and its low-serum growth supplement (LSGS) (hereafter referred as M200), and autophagy failed to neutralize invading GAS under this condition (15). In the present study, we initially used EGM-2MV medium instead (hereafter referred as EGM2). Unexpectedly, the number of GAS organisms (NZ131, serotype: M49) that survived in endothelial cells was much lower after culture in EGM2 compared to M200 (Fig. 1A; Fig. S1A). EGM2 contains three ingredients that are not present in M200, namely, ascorbic acid, R3-insulin-like growth factor-1, and VEGF. Each was separately removed from EGM2 to evaluate its effect on GAS proliferation within endothelial cells. The results showed that GAS proliferation in EGM2 was not suppressed in the absence of VEGF (Fig. 1B; Fig. S1B), which was also the case in M200. VEGF neither directly inhibited GAS growth nor caused defective expression of the GAS virulence factors SLO and SLS (Fig. S1C and D), which enable GAS to escape from the endosomal membrane into the cytosol (8). In addition, adding VEGF to M200 suppressed GAS proliferation, and the effect was abolished by the administration of the VEGF receptor inhibitor axitinib (Fig. 1C; Fig. S1E). VEGF suppression of intracellular GAS growth was also observed in the M1 serotype A20 GAS strain (Fig. S1F). The VEGF level in culture supernatant without exogenous administration of VEGF was around 1 ng/mL. This level was presumably achieved by autocrine secretion, and was around 10 times lower than that following VEGF administration. It should be sufficient for cellular growth but not for an antibacterial effect. These results indicate that augmentation of the VEGF-dependent signaling pathway enhances GAS clearance inside endothelial cells.

10.1128/mbio.01233-22.1 FIG S1 VEGF had no direct effect on GAS growth or virulence factors, but promoted xenophagy to suppress GAS growth in endothelial cells. (A and B) CFAs were performed as described in Fig. 1A and B, and the data represent the GAS colony number determined at 1-h (A and B) and 6-h (A) postinfection. There is no significant difference in GAS internalizing efficacy between groups. (C) GAS broth cultured overnight was diluted 50-fold in fresh broth with or without 1 nM VEGF, and cultured at 37°C for the indicated durations. The absorbance of GAS culture broth was measured at 600 nm by a spectrophotometer. (D) The hemolytic activity of GAS culture supernatants was measured. (E) CFAs were performed as described in Fig. 1C, and the data represent the GAS colony number determined at 1-h postinfection. (F) HMEC-1 cells treated with or without VEGF were infected with A20 GAS for CFAs. Bacterial preparation of the A20 strain for CFAs was performed using the same protocol as that for the NZ131 strain. (G) HMEC-1 cells expressing GFP-LC3 and mStrawberry-Gal-3 were treated with VEGF for 2 d and infected with GAS for 1 h. Samples were fixed and stained with Hoechst, and GAS clusters positive for GFP-LC3 were observed under a confocal microscope. Arrowheads indicate double-membrane structures, and the arrow indicates an autophagolysosome (AL) characterized by a multimembrane structure. Scale bar: 1 μm. (H) VEGF-treated wild-type or ATG7 KO HMEC-1 cells were infected with GAS, and the numbers of intracellular GAS organisms were determined by CFAs. Fold increase in growth was calculated by the colony number at 6 h normalized to that at 1-h postinfection. Experiments were repeated at least twice, and the data from one set of experiments performed in triplicate are shown. (I) HMEC-1 cells treated with or without VEGF were infected with GAS for 1 h and then fixed for electronic microscopy sample preparation. L, lysosome; AL, autophagolysosome. Scale bar, 1 μm. Download FIG S1, PDF file, 0.7 MB. Copyright © 2022 Lu et al. 2022 Lu et al. https://creativecommons.org/licenses/by/4.0/ This content is distributed under the terms of the Creative Commons Attribution 4.0 International license.

### VEGF promoted xenophagy against GAS in endothelial cells.

To investigate the mechanism whereby VEGF suppressed GAS proliferation, we first observed the intracellular distribution of GAS that had invaded endothelial cells. Without VEGF treatment in M200, GAS showed a widespread distribution (Fig. 1D, black arrowheads), while VEGF treatment caused GAS to form packed clusters (Fig. 1D, red arrowheads). VEGF treatment resulted in more effective recruitment of LC3, the marker used to identify autophagosomes (22) (Fig. 1E and F). On the other hand, we and other groups previously reported that an LC3-positive signal does not necessarily represent the typical double-membrane structure of autophagosomes, but may also indicate another membrane structure called the LC3-associated phagosome (LAPosome) (15, 23, 24). In phagocytes, the pH of LAPosomes is reduced to a low level that effectively kills bacteria. However, LAPosomes in endothelial cells are unable to maintain acidification, leading to failure of GAS growth suppression (15). In addition to LAPosomes, canonical autophagosomes (with a double-membrane structure) are critical for bacterial clearance (15, 25). To distinguish between the structures of canonical autophagosomes and LAPosomes, we analyzed LC3-positive GAS structures by correlative light electron microscopy (CLEM). In nontreated cells, GFP-LC3-positive GAS were positive for mStrawberry-galectin -3 (Gal-3), a lectin that is a marker of damaged endosomes/lysosomes (26); the double-positive structures only exhibited a single membrane, indicating that they were LAPosomes (Fig. S1G), which is consistent with our previous findings (15). In contrast, GFP-LC3 single-positive GAS in VEGF-treated cells showed double-membrane structures representing autophagosomes (Fig. S1G). Conventional transmission electron microscopy also exhibited clear double-membrane structures representing isolation membranes (IMs), which are precursors of autophagosomes, in the VEGF-treated group, while only single-membrane structures were observed in nontreated cells (Fig. 1G). The other criterion for distinguishing between autophagosomes and LAPosomes is recruitment to GAS of FIP200, a key protein for autophagosome formation (24). LC3-positive GAS was negative for FIP200 in nontreated cells, while VEGF treatment promoted FIP200 recruitment to LC3-positive GAS (Fig. 1H and I). These data indicate that VEGF treatment promotes autophagy targeting of GAS (xenophagy) in endothelial cells.

To determine whether VEGF-dependent autophagy induction is involved in suppression of GAS proliferation, we compared the number of GAS that survived in wild-type endothelial cells versus cells knocked out for ATG7 (ATG7 KO), an essential protein for autophagy (27, 28). GAS survival was higher in ATG7 KO cells than in wild-type cells, indicating that autophagy plays a role in suppressing GAS proliferation (Fig. S1H). Meanwhile, dose-dependent VEGF-mediated suppression of GAS proliferation was observed even in autophagy-deficient cells (Fig. 1J). This implies that VEGF promotes bacterial clearance not only via xenophagy but also through other pathways.

### VEGF enhanced lysosomal function against bacteria in endothelial cells.

**FIG 2:**
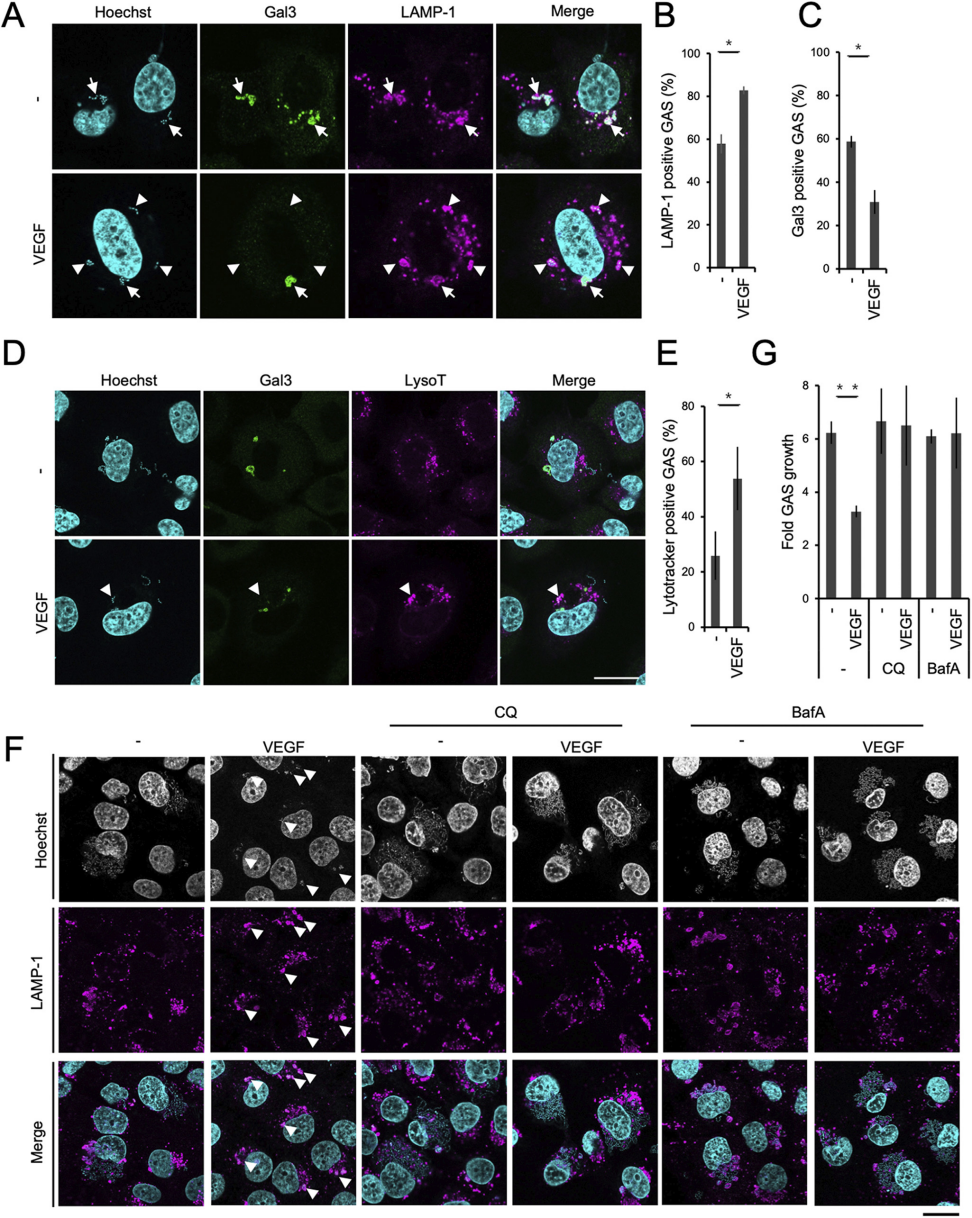
VEGF treatment boosted lysosomal function in endothelial cells. HMEC-1 cells were incubated in M200 with or without VEGF at 1 nM for 2 d. GAS was used to infect endothelial cells for 1 h, and GAS-infected cells were fixed and stained with anti-Gal-3 (A and D) and anti-LAMP-1 (A) antibodies. (D) HMEC-1 cells were prestained with 100 nM Lysotracker 30 min before GAS infection. GAS was stained by Hoechst. (B, C, and E) The graph represents the percentage of GAS that are marker positive in three independent experiments. The number of GAS collected was over 100 in each sample. (F) Cells were treated with or without VEGF and infected with GAS. After 30-min bacterial inoculation, VEGF was added along with either chloroquine (50 μM) or bafilomycin A1 (200 nM) and antibiotic until 6-h postinfection. (G) Cells were treated as in (F) and CFAs were performed at 1 h and 6 h to calculate the fold increase in GAS replication. White arrowheads in (F) indicate small GAS clusters trapped in lysosomes (LAMP-1). The graphs represent the mean and SD from three independent experiments. Scale bars are 10 μm. *, *P < *0.05; **, *P < *0.01 by unpaired two-tailed Student’s *t* test.

To identify the aforementioned autophagy-independent pathway, we focused on lysosomal function, because electron microscopy images showed that large numbers of lysosomes were located near GAS vacuoles, but only in VEGF-treated cells (Fig. S1I). VEGF did in fact increase the recruitment of LAMP-1, a lysosome marker, to GAS (Fig. 2A and B). Notably, a significant proportion of LAMP-1-positive GAS did not exhibit Gal-3, suggesting that GAS-derived hemolysin activity was suppressed (Fig. 2A and C). Previous reports showed that low pH conditions, such as those within lysosomes, decreased the generation and secretion of SLO and SLS (14), and even stopped GAS replication (29). We analyzed the pH of endosomes/lysosomes containing GAS using Lysotracker, a pH-sensitive probe. VEGF treatment increased the number of Lysotracker-positive, GAS-containing vacuoles, indicating that pH was significantly lowered (Fig. 2D and E). GAS in VEGF-treated cells could not proliferate and were effectively trapped within lysosomes (Fig. 2F), while treatment with chloroquine or bafilomycin A_1_, both of which increase lysosomal pH (Fig. S2A to D), canceled these VEGF-dependent effects and yielded results that were comparable to the nontreated condition (Fig. 2F and G; Fig. S2E). These findings indicate that VEGF promotes lysosomal activity that eliminates GAS.

10.1128/mbio.01233-22.2 FIG S2 Chloroquine decreased intracellular vesicle acidification and did not directly affect GAS growth *in vitro*. (A, B) HMEC-1 cells were treated with 50 μM chloroquine (CQ) for 1 h or 6 h, then 100 nM Lysotracker was added to stain lysosomes for 30 min, and cells were fixed. Samples were observed under a confocal microscope (A), and cytosolic fluorescence was measured by ImageJ (B). Thirty cells were detected in each group. Scale bar, 10 μm. (C and D) GAS broth cultured overnight was diluted 50-fold in fresh broth with or without 50 μM chloroquine, then cultured at 37°C for the indicated durations. GAS growth was measured by spectrophotometer (C). After culture for the indicated durations, GAS culture broth was serially diluted and plated on agar, and colony numbers were determined after 1-d incubation (D). (E) CFAs were performed as described in Fig. 2G, and the data represent the GAS colony number determined at 1-h and 6-h postinfection. Experiments were repeated twice, and the data from one set of experiment performed in triplicate are shown. Download FIG S2, PDF file, 0.3 MB. Copyright © 2022 Lu et al. 2022 Lu et al. https://creativecommons.org/licenses/by/4.0/ This content is distributed under the terms of the Creative Commons Attribution 4.0 International license.

### VEGF activates TFEB and increases lysosomal biogenesis.

**FIG 3:**
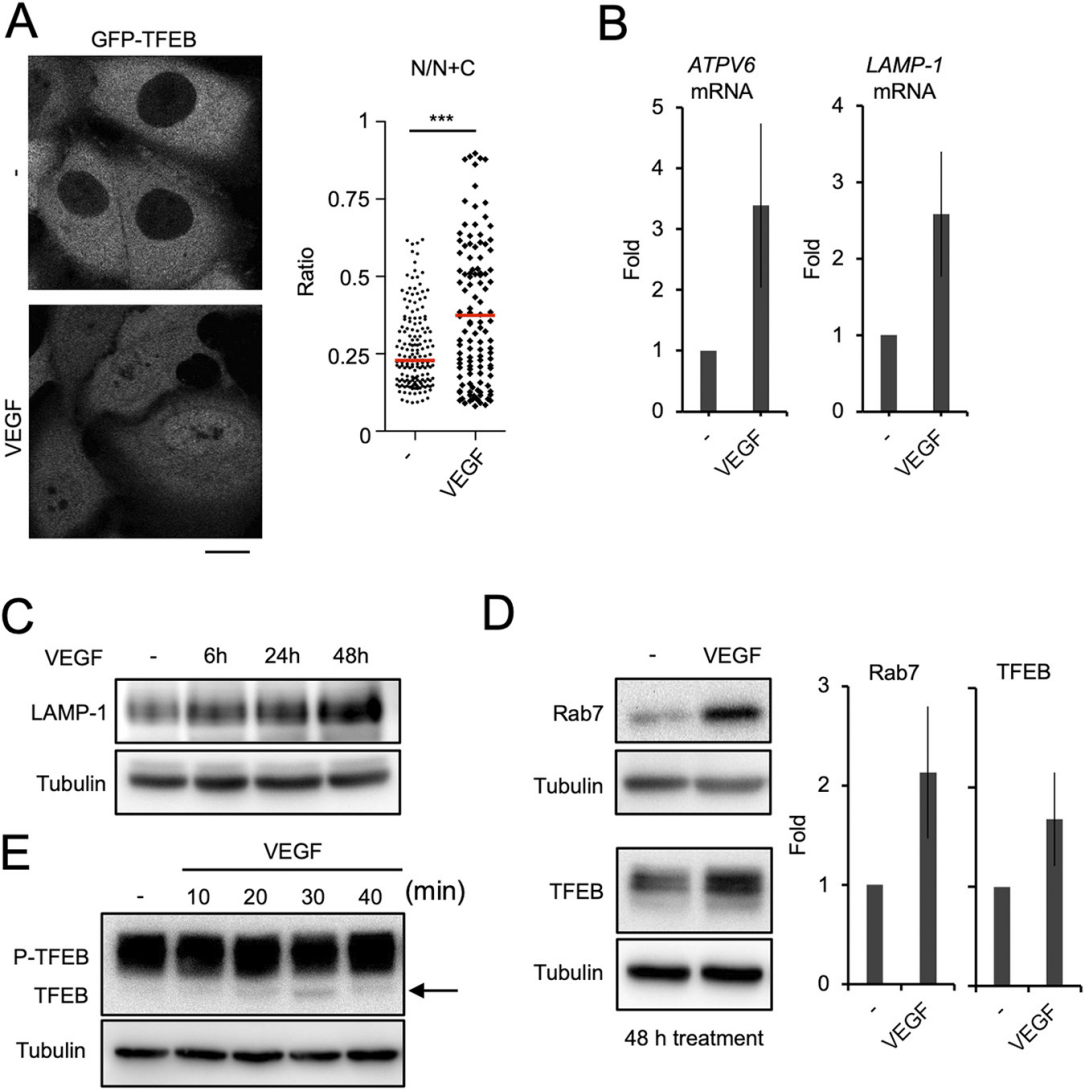
VEGF treatment activated TFEB-dependent lysosomal biogenesis. (A) HMEC-1 cells expressing GFP-TFEB were treated with VEGF for 1 h. N/N+C, ratio of mean fluorescence intensity of the nucleus to that of the nucleus plus cytosol. Scale bars are 10 μm. (B-E) HMEC-1 cells were treated with or without VEGF for periods differing by 10-min intervals (E), or for 48 h (B, D) or 6, 24, or 48 h (C), and the indicated markers of mRNA (B) or proteins (C, D, and E) were determined by q-PCR or Western blot assay. Experiments were repeated at least three times. Data represent the mean and SD from three independent experiments. *, *P < *0.05; **, *P < *0.01 by unpaired two-tailed Student’s *t* test.

The expression of genes related to lysosomal biogenesis is regulated by transcription factor EB (TFEB), which is activated and then enters the nucleus in response to various environmental stimuli (30). We found that VEGF stimulation of endothelial cells promoted TFEB nuclear translocation (Fig. 3A). Accordingly, the expressions of TFEB downstream genes, including *ATPV6* and *LAMP-1*, were upregulated by VEGF treatment (Fig. 3B). In addition, the amounts of the lysosomal proteins LAMP-1, Rab7, and even TFEB itself were significantly increased (Fig. 3C and D). These results indicate that VEGF can activate the TFEB pathway in endothelial cells.

**FIG 4:**
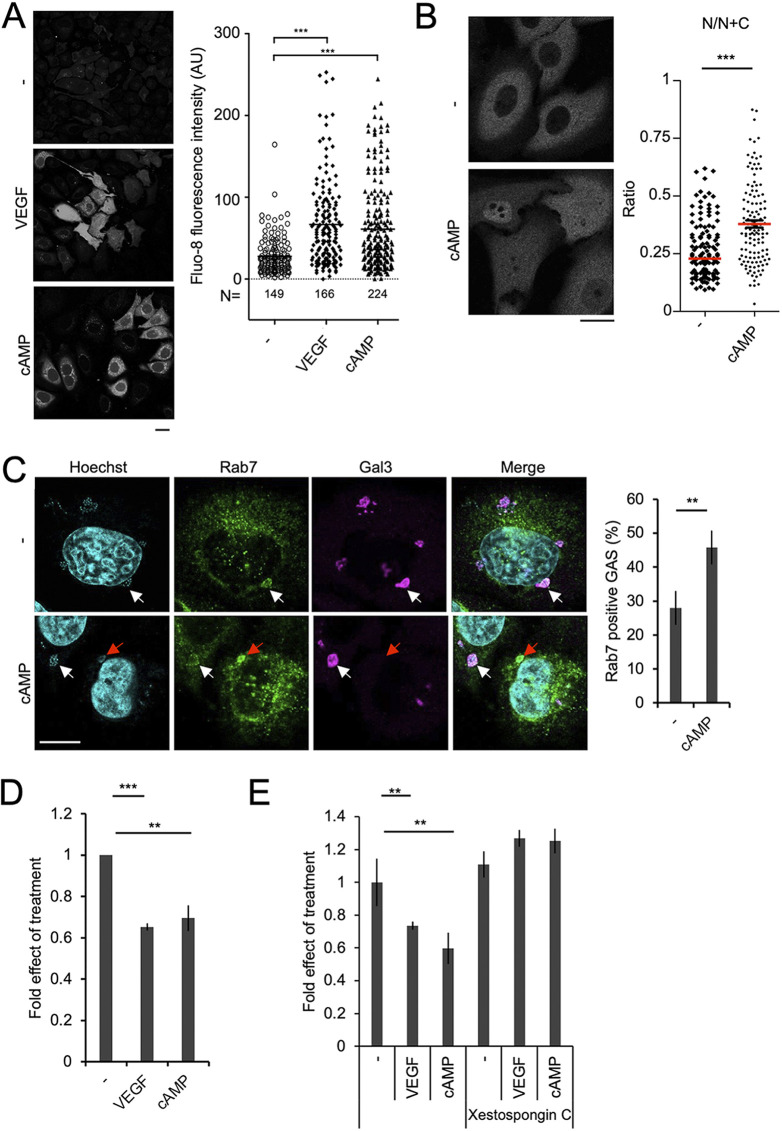
VEGF-mediated GAS suppression through the cAMP-IP_3_-Ca^2+^-dependent pathway. (A) To measure the cytosolic Ca^2+^ concentration, HMEC-1 cells were treated with VEGF or 100 μM dibutyryl cAMP for 2 d and then stained with Fluo-8 for 1 h. Cells were again treated with VEGF or cAMP after washout of Fluo-8. Images were captured at 1-h post-treatment and the fluorescence intensity of an area of cytosol was measured by Image J. N indicates the measured cell numbers in each sample. (B) HMEC-1 cells expressing GFP-TFEB were treated with 100 μM dibutyryl cAMP for 1 h and then observed under a confocal microscope. N/N+C, ratio of mean fluorescence intensity of the nucleus to that of the nucleus plus cytosol. (C) HMEC-1 cells treated with dibutyryl cAMP were infected with GAS for 1 h and then stained with anti-Rab7, Gal-3, and Hoechst. White arrows, Rab7- and Gal3-positive GAS; red arrows, Rab7-positive GAS. Over 100 GAS organisms were observed in each sample. (D, E) HMEC-1 cells were treated with dibutyryl cAMP or VEGF for 2 days and infected with GAS for 1 h and 6 h for CFA assays. The fold increase in GAS growth was calculated, and the fold of effect from VEGF or cAMP treatment was further normalized to the control group. (E) Xestospongin C was added along with gentamicin at 30 min after GAS inoculation. Data represent the mean and SD from three independent experiments. Scale bars are 10 μm. **, *P < *0.01; ***, *P < *0.001 by unpaired two-tailed Student’s *t* test.

TFEB is restricted to the cytoplasm when it is phosphorylated by the mTORC1 protein kinase. However, once mTORC1 is inactivated, the calcium-dependent phosphatase calcineurin dephosphorylates TFEB, causing it to enter the nucleus (31). First, we examined whether VEGF affected the phosphorylation of the mTORC1 substrates TFEB, S6 kinase, and 4E-BP1. VEGF slightly attenuated mTORC1 activity, as shown by the dephosphorylation of TFEB (Fig. 3E) as well as S6 kinase and 4E-BP1 (Fig. S3A). Because the effect was not pronounced, we next focused on the Ca^2+^-dependent pathway. Previous reports showed that VEGF stimulation induced Ca^2+^ release into the cytosol (32, 33). Cyclic AMP (cAMP) functions as the downstream signaling molecule of the VEGF receptor (34). It mediates IP_3_ release from the plasma membrane, which activates IP_3_ receptors on the ER, resulting in ER Ca^2+^ release (35). Here, we showed that VEGF treatment increased the cytosolic concentration of Ca^2+^ (Fig. 4A). Furthermore, treatment with dibutyryl cAMP, a cell-permeable cAMP analogue, also increased cytosolic Ca^2+^ (Fig. 4A). As with VEGF stimulation, treatment with dibutyryl cAMP induced nuclear translocation of TFEB (Fig. 4B). It also promoted Rab7 targeting of GAS-containing endosomes whose membranes were Gal-3-negative, indicating lack of injury (red arrow) (Fig. 4C). Furthermore, dibutyryl cAMP treatment suppressed GAS growth in endothelial cells to a comparable degree as VEGF treatment (Fig. 4D; Fig. S3B). Most importantly, xestospongin C, an inhibitor of IP_3_ receptors that blocks Ca^2+^ release from the ER, canceled VEGF- and dibutyryl cAMP-dependent suppression of GAS growth (Fig. 4E). These results indicate that VEGF-mediated Ca^2+^ signaling leads to TFEB activation and might link to the suppression of GAS growth in endothelial cells.

10.1128/mbio.01233-22.3 FIG S3 VEGF slightly modulated mTORC1 activity and the stimulation of VEGF signaling had no effect on GAS internalization. (A) HMEC-1 cells were treated with or without VEGF for 40 min and cells were collected for western blot analysis. The phosphorylation of mTOR substrates, namely ULK1, S6 kinase, and 4E-BP1, and of total forms of S6 kinase and internal control tubulin, was determined. The experiments were repeated at least three times, and data are presented as the ratio of the intensity of each target protein to that of internal control. (B) CFAs were performed as described in Fig. 4D, and the data from one set of experiments represents the GAS colony number determined at 1-h postinfection. There is no significant difference in GAS-invasion efficacy between groups. Download FIG S3, PDF file, 0.04 MB. Copyright © 2022 Lu et al. 2022 Lu et al. https://creativecommons.org/licenses/by/4.0/ This content is distributed under the terms of the Creative Commons Attribution 4.0 International license.

### TFEB activation occurs during defense against GAS infection.

**FIG 5:**
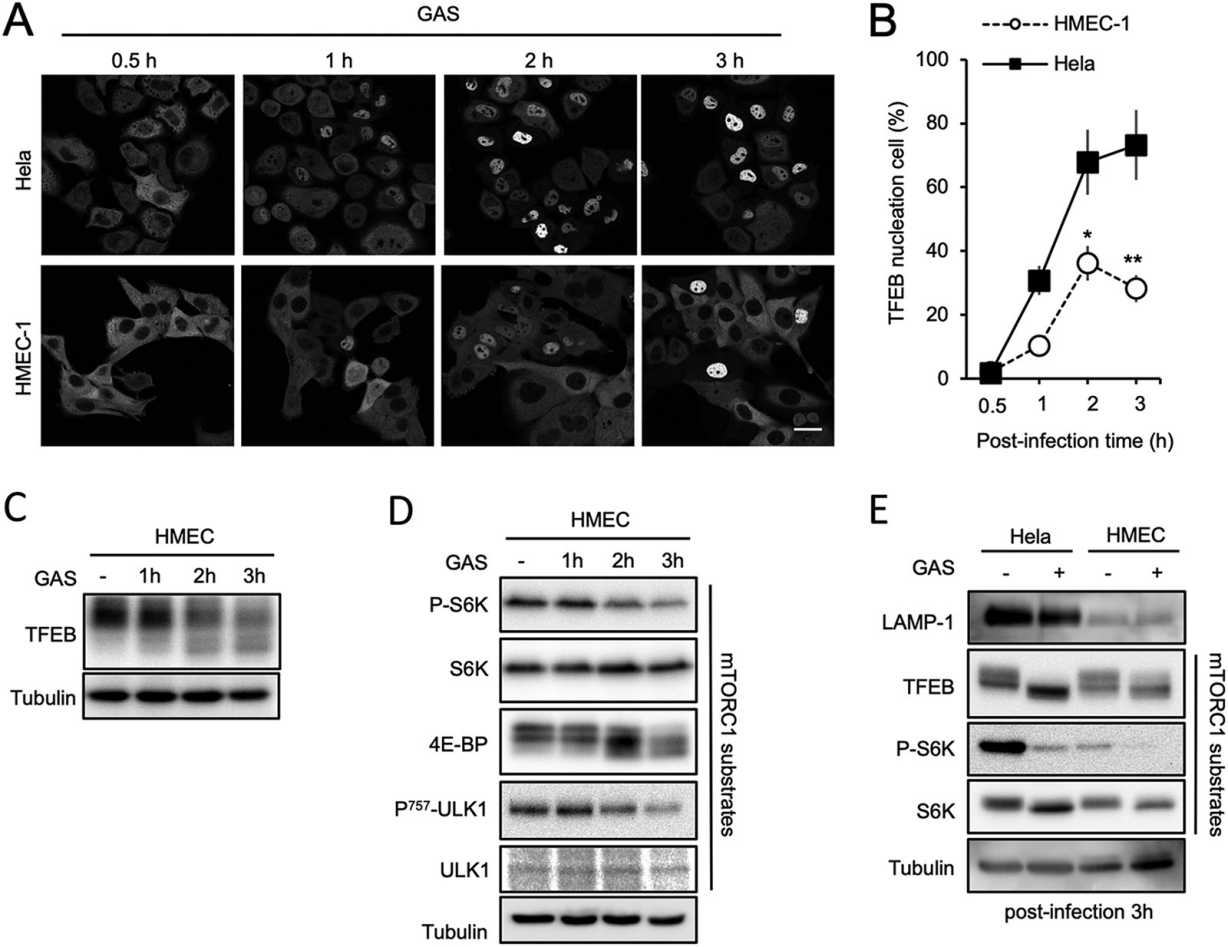
GAS infection suppressed mTORC1 activity and induced TFEB nuclear translocation. (A) To infect an equal engulfed bacterial number, HeLa cells or HMEC-1 cells expressing GFP-TFEB were added with GAS at MOI 25 or 5, respectively, and fixed at the indicated time points. Antibiotic was continually present in the culture medium to kill extracellular GAS after 30-min bacterial inoculation. (B) For each cell in (A), if the fluorescence intensity in the nucleus was equal to or higher than that in the cytosol, the cell was counted as positive for TFEB nuclear translocation (N/N+C ≥ 0.5). The number of positive cells was normalized to the number of GAS-infected cells. HMEC-1 cells were infected with GAS for 1, 2, or 3 h (C, D), and HMEC-1 and HeLa were infected with GAS for 3 h (E). The phosphorylation of each protein was detected by Western blot analysis. Experiments were repeated as least twice, and representative blots are shown.

Although TFEB is known to play a regulatory role in lysosomal biogenesis, its contribution to the cellular response during GAS infection is still unknown. Here, we found that GAS infection induced TFEB nuclear translocation in HMEC-1 endothelial cells (Fig. 5A). Furthermore, TFEB nuclear translocation occurred to a much greater extent in epithelial HeLa cells than in endothelial cells (Fig. 5A and B). In addition, GAS infection of endothelial cells resulted in time-dependent TFEB dephosphorylation (Fig. 5C). Because the mTORC1 substrates S6K, 4E-BP1, and ULK1 were also dephosphorylated, mTORC1 was presumably suppressed by GAS infection (Fig. 5D). However, these mTORC1 substrates were dephosphorylated to a greater extent in epithelial cells than in endothelial cells (Fig. 5E). The expression level of the lysosomal protein LAMP-1 was consistently much higher in epithelial cells than in endothelial cells (Fig. 5E). These results suggest that the mTORC1-TFEB pathway is correlated with GAS clearance.

### VEGF-mediated TFEB activation enhanced GAS clearance in endothelial cells.

**FIG 6:**
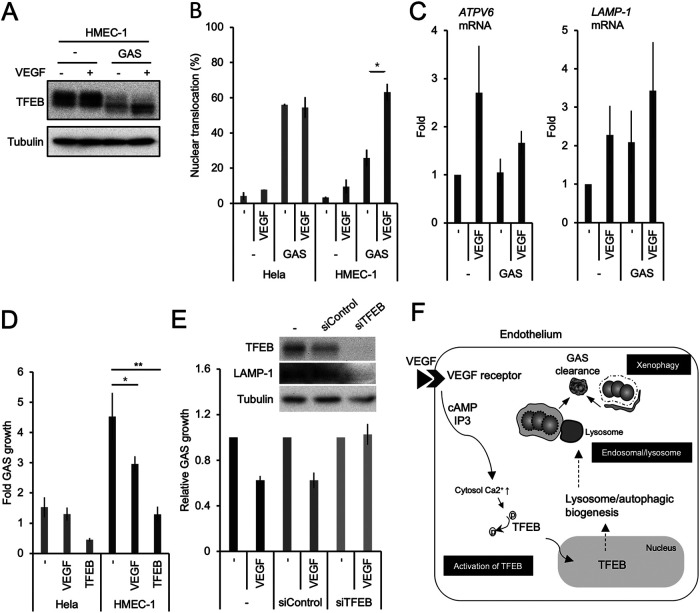
VEGF-mediated TFEB activation increased GAS clearance in endothelial cells. (A) HMEC-1 cells were treated with VEGF for 2 d and infected with GAS for 3 h. Cells were collected for Western blot analysis. (B) HMEC-1 cells expressing GFP-TFEB were treated with or without VEGF and then infected with GAS for 1.5 h. The percentage of TFEB nuclear translocation was calculated as in Fig. 5A and B. (C) VEGF-treated HMEC-1 cells were infected with GAS for 24 h, and mRNA were subjected to q-PCR. (D) CFAs were performed to detect GAS growth in HeLa and HMEC-1 cells with or without TFEB overexpression and VEGF treatment. (E) HMEC-1 cells were transfected with TFEB siRNA twice and were treated with or without VEGF. TFEB and LAMP-1 proteins were confirmed by Western blot analysis, and CFA assays were performed under the same conditions. All quantitative data represent the mean and SD from three independent experiments. *, *P < *0.05; **, *P < *0.01 by unpaired two-tailed Student’s *t* test. (F) The molecular mechanism of VEGF-mediated antimicrobial activity *in vitro*.

Next, we hypothesized that the weak TFEB dephosphorylation in GAS-infected endothelial cells might be mitigated by VEGF treatment. Indeed, VEGF treatment resulted in greater dephosphorylation of TFEB in GAS-infected endothelial cells (Fig. 6A). Furthermore, VEGF enhanced the efficacy of nuclear translocation of TFEB in GAS-infected endothelial cells, to an extent that was comparable with that in epithelial cells (Fig. 6B). The expressions of the TFEB downstream genes *ATPV6* and *LAMP-1* were also upregulated by VEGF following GAS infection (Fig. 6C). In addition, the overexpression of TFEB suppressed GAS growth in endothelial cells (Fig. 6D). In contrast, knockdown of TFEB by siRNA canceled VEGF-dependent suppression of GAS growth (Fig. 6E). These data imply that VEGF suppression of bacterial growth may be mediated by enhancement of the TFEB pathway. Taken together, VEGF exerts an antimicrobial effect in endothelial cells *in vitro* (Fig. 6F).

### VEGF signaling was correlated with the severity of GAS infection *in vivo*.

**FIG 7:**
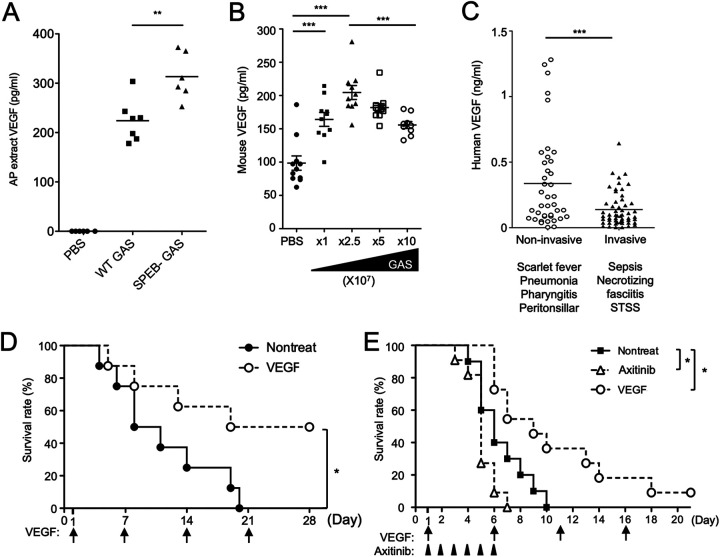
Evaluation of the effect of VEGF on GAS infection *in vivo*. (A) In an air pouch infection model, mice were infected with 2 mL of air in the subcutaneous skin of the back, followed by injection with GAS at 2 × 10^8^ CFU in 200 μL PBS. At 24-h postinfection, air pouch extracts were collected by rinsing with 1 mL PBS and withdrawing the liquid after 5-s massage. (B) Mice were infected with GAS at the indicated doses through tail vein injection. At 24-h postinfection, whole blood was collected. Mouse VEGF concentrations in sera or air pouch extracts were determined by ELISA. Each dot represents one mouse. (C) VEGF concentrations in sera collected from GAS-infected human patients as determined by ELISA. Patients were separated into noninvasive and invasive disease groups according to their clinical diagnoses, as described in the “Patient sera” section of the Materials and Methods. (D) The survival rates of GAS-infected mice following VEGF treatment. Mice were infected with GAS at 5 × 10^7^ CFU through tail vein injection. Subsequently, 2 μg of VEGF per mouse was administered through tail vein injection 1-d postinfection and again on days 7, 14, and 21 postinfection. Each group contained eight mice. (E) The survival rates of mice infected with GAS and then treated with VEGF or axitinib. Mice were infected with GAS at 1 × 10^8^ CFU through tail vein injection. Then, 2 μg of VEGF per mouse was administered through tail vein injection 1-d postinfection and again every 5 d thereafter. In the experimental group, 1 mg of axitinib per mouse was orally administered every day postinfection. Each group contained 10 mice. The survival rate is displayed as a Kaplan-Meier survival curve and assessed by the log-rank test. *P* values < 0.05 indicate significant differences (GraphPad Prism software version 5).

We next examined the relationship between VEGF and GAS infection *in vivo*. We investigated changes in VEGF levels in GAS-infected mice. In a local skin infection model, GAS was inoculated into subcutaneous tissue within an air pouch. Twenty-four hours later, we confirmed that bacteremia had not yet occurred at this early stage. We analyzed air pouch extracts and found that VEGF levels had increased significantly at the local infection site (Fig. 7A; Fig. S5A). Even though extracts were diluted in 1 mL of phosphate-buffered saline (PBS) buffer, the VEGF level reached a mean of 224 pg/mL in the wild-type GAS group, and thus the actual level was much higher.

10.1128/mbio.01233-22.5 Figure S5 VEGF and sVEGFR1 levels in mouse infection models or GAS patients. (A) In the air pouch infection model described in Fig. 7A, mice were infected with 2 mL of air in the subcutaneous skin of the back, followed by injection with WT or SpeB mutant GAS at 2 × 10^8^ CFU in 200 μL PBS. At 24-h postinfection, whole blood was collected. Mouse VEGF levels in serum were determined by ELISA (DY493-05 for mouse VEGF, R&D Systems). Each dot represents one mouse. There is no significant difference between the three groups. (B) VEGF levels in the patient sera (87 in total) characterized in Fig. 7C were further analyzed according to patient age. High VEGF concentration is correlated with younger age in patients with non-invasive disease but not in those with invasive disease. (C) Soluble VEGFR1 levels in sera in a GAS-induced sepsis model. Mice were infected with GAS at the indicated doses through tail vein injection. At 24-h postinfection, whole blood was collected. Mouse soluble VEGFR1 levels in sera were determined by ELISA (MVR100-1 for sVEGFR1, R&D Systems). Each dot represents one mouse. (D) In the air pouch infection model described in Fig. 7A, air pouch extracts were collected by rinsing with 1 mL PBS and withdrawing the liquid after 5-s massage. The infiltrating immune cells in the air pouch extract were stained by trypan blue and only the live cells were counted. *, *P < *0.05; **, *P < *0.01; ***, *P < *0.001 by unpaired two-tailed Student’s *t*-test. Download FIG S5, PDF file, 0.10 MB. Copyright © 2022 Lu et al. 2022 Lu et al. https://creativecommons.org/licenses/by/4.0/ This content is distributed under the terms of the Creative Commons Attribution 4.0 International license.

SpeB is a cysteine protease virulence factor that plays a critical role in the pathogenesis of local infectious disease (36), and SpeB-deficient GAS further increased VEGF levels at the local infection site compared with wild-type GAS (Fig. 7A), although SpeB in GAS culture supernatant barely degraded VEGF (Fig. S4).

10.1128/mbio.01233-22.4 FIG S4 SpeB did not directly degrade VEGF. A 100-μL volume was taken from the broth of wild-type (A to C) or SpeB mutant (B) GAS cultured overnight, then added to 40 ng human VEGF recombinant protein generated by Sf21 baculovirus vector (293-VE, R&D Systems) or E. coli (100-20, PeproTech), and further incubated at 37°C for 6 h (A, B) or 24 h (C). GAS particles were removed by centrifugation at 3,500 rpm and the supernatant was subjected to western blot analysis using anti-human VEGF165 antibody (BS-0279R, Bioss). The expected molecular size of VEGF monomers was around 19.2 kDa. Download FIG S4, PDF file, 0.06 MB. Copyright © 2022 Lu et al. 2022 Lu et al. https://creativecommons.org/licenses/by/4.0/ This content is distributed under the terms of the Creative Commons Attribution 4.0 International license.

We next measured serum VEGF levels in a mouse model of GAS-induced sepsis. We found that VEGF levels were increased following infection with low-dose GAS administered by tail vein injection (Fig. 7B), which was expected due to the immune inflammatory response (37). However, once GAS reached lethal doses (5 × 10^7^ and 10^8^ CFU/mouse), VEGF levels decreased (Fig. 7B). A similar trend was also observed in human patients infected with GAS. Patients were divided into noninvasive and invasive disease groups according to their symptomatology. Patients with severe symptoms indicative of bacterial invasion, such as bacteremia, sepsis, and necrotizing fasciitis, exhibited lower serum VEGF levels than those with symptoms not characterized by invasion (Fig. 7C). The low VEGF levels in the invasive disease group were not associated with patient age (Fig. S5B).

A previous report showed that nonsurviving patients with severe sepsis and multiple organ failure had lower VEGF levels and higher soluble VEGF receptor 1 (sVEGFR1) levels than surviving patients (38). sVEGFR1 functions as a competitive inhibitor by binding with VEGF to reduce the interaction of VEGF-VEGFR1 on endothelial cells (39). Our mouse model of sepsis also showed the same pattern: mice infected with lethal doses of GAS had lower VEGF levels and higher sVEGFR1 levels than those infected with nonlethal doses (Fig. S5C). Thus, the severity of GAS infection symptoms seems to be correlated with the strength of VEGF signaling.

To determine whether higher levels of VEGF signaling protect the host from GAS infection *in vivo*, we analyzed mortality associated with different levels of VEGF signaling in a sepsis model involving lethal doses of GAS. After systemically infecting mice with GAS at 5 × 10^7^ CFU (CFU), we intravenously injected VEGF on days 1, 7, 14, and 21. VEGF treatment significantly increased the mouse survival rate (Fig. 7D). A similar effect was observed with a higher number of GAS organisms (1 × 10^8^ CFU) (Fig. 7E). Furthermore, mouse mortality was increased when the VEGF signaling pathway was blocked by the oral administration of axitinib, a VEGF receptor inhibitor, at a dose that did not affect mouse survival in the absence of GAS infection (Fig. 7E) (40). These data suggest that stronger VEGF signaling may help GAS-infected mice overcome acute infection.

## DISCUSSION

In this study, we revealed that VEGF potentiated lysosomal and autophagic functions that defend against GAS infection in endothelial cells (Fig. 6F). Activation of the VEGF signaling pathway also protected against GAS infection *in vivo*. Furthermore, severe symptoms of GAS infection were accompanied by diminished serum VEGF levels. Therefore, VEGF may be a key factor affecting the susceptibility of endothelial cells to GAS infection.

In comparison with epithelial cells, endothelial cells have limited steady-state autophagic and lysosomal capacity against GAS. While epithelial cells are continually exposed to external pathogens and commensal microbiota, endothelial cells are basically quarantined in a relatively sterile environment. To reduce energy expenditure, therefore, it is reasonable for the latter to maintain a limited basal capacity for autophagic and lysosomal targeting of GAS, and to boost this capacity via VEGF when necessary. It has been reported that the autophagy modulator acacetin increases TFEB activation through an mTORC1-independent pathway, and restricts intracellular Salmonella replication (41). Here, we found that VEGF amplified autophagic and lysosomal degradation, at least partly through the TFEB pathway. The cAMP-IP_3_-Ca^2+^ axis seems to be primarily responsible for signal transmission from VEGF to TFEB, and this axis does not increase ubiquitination in endothelial cells (data not shown) (42). However, GAS infection itself upregulates the TFEB pathway, primarily through the mTORC1 axis. Especially in endothelial cells, the combination of these two axes seems to boost autophagic and lysosomal targeting of GAS. Although it remains unclear how GAS infection suppresses mTORC1-dependent processes, previous reports have shown that damaged lysosomes release mTORC1 complexes that suppress kinase activity (43), and lysosomal release of Ca^2+^ results in dephosphorylation of TFEB (44).

We administered 2 μg VEGF intravenously in blood circulation per mouse, as shown in Fig. 7D and E, but this dose did not increase vessel permeability throughout the body (data not shown), which would be expected if VEGF were administered at a high enough dose. However, even at such low doses, VEGF administration significantly increased mouse survival following GAS infection (Fig. 7D). The dose of 2 μg VEGF per mouse is estimated to lead to a concentration of roughly 50 nM in the total blood volume, while the concentration of VEGF we administered *in vitro* was 1 nM (19.2 ng/mL), which is higher than the endogenous VEGF concentration in mouse blood (Fig. 7B). It has been reported that macrophages are recruited to sites of bacterial infection and secrete VEGF to locally enhance vascular permeability, which enables recruitment of additional immune cells (45). Therefore, local VEGF concentrations following GAS infection of endothelial cells are postulated to be high enough to exert autophagic and lysosomal effects. We also showed that VEGF levels at local infection sites in air pouch infectious model were much higher than in sera in a sepsis model (Fig. 7A and B). We examined the possibility that SpeB directly degrades VEGF proteins, but did not identify significant degradative activity (Fig. S4). Previous reports showed that SpeB directly damages tissue and induces immune cell death (36, 46). Consistent with these findings, we showed that the number of immune cells at the site of GAS infection was lower in wild-type mice than in those with SpeB mutant GAS (Fig. S5D). This may cause the reduced VEGF levels secreted from the immune cells of wild-type mice following GAS infection.

While local VEGF levels are high at the site of a local skin infection, once bacteria disseminate into blood vessels, endogenous VEGF levels in the blood may be far too low to stimulate intracellular killing of GAS, although there is limited evidence that circulating GAS can directly invade blood vessel endothelial cells *in vivo*. The results from our animal model of sepsis showed that administering VEGF and thereby increasing its concentration in the circulation could overcome sepsis-mediated death, which is associated with blood vessel injury, fluid leakage, hypotension, and then septic shock (47).

Based on our findings in this study, we propose a model regarding the establishment of GAS infection. Once GAS infects endothelial cells by disrupting several physical and immunological barriers, including skin and vessels, neighboring macrophages secrete VEGF that potentiates the autophagic and lysosomal GAS killing activity of endothelial cells. However, if this potentiation fails to occur effectively due to reduced VEGF levels and enhanced sVEGFR1 levels, the elimination of GAS will be unsuccessful and infectious symptoms will be severe. GAS is capable of invading nonimmune cells to escape phagocytosis and the effects of antibiotics. The intracellular innate immune system of host cells, including the autophagic and lysosomal degradation system, should be activated along with the adaptive immune system. Recent reports have shown that immune regulation is an alternative to antibiotic therapy for the treatment of sepsis (48). Our theory suggests the potential for other new and effective therapies. One possibility is the local or systemic administration of recombinant VEGF protein to the affected areas of patients with GAS infection. VEGF is required for wound repair (49), and VEGF concentrations in septic patients usually correlate with proper healing (50). A recent paper further reported that high expression of VEGF extend the life span of mice by reducing visceral fat deposition and maintaining microvascular density (18). It is known that autophagy and lysosomal functions are tightly associated with aging and longevity (51). Here, we showed that VEGF activates TFEB to promote autophagic/lysosomal functions, and hypothesize that this mechanism may directly link VEGF and aging. Administration of exogenous VEGF will provide an avenue for the future treatment of patients with bacteremia and potentially increase longevity.

## MATERIALS AND METHODS

### Cell culture.

Human microvascular endothelial cell line-1 (HMEC-1) (52) (obtained from the Centers for Disease Control and Prevention, USA) was cultured in endothelial cell growth medium M200 (M200-500, Invitrogen) containing LSGS supplement (S-003-10, Invitrogen). The other endothelial cell medium set used was the EGM-2MV set (CC-3202, Lonza). Both M200 and EGM2 media were adjusted to contain 10% fetal bovine serum (FBS). ATG7-knockout HMEC-1 generated by a CRISPR-Cas9 system and GFP-LC3-expressing HMEC-1 cells generated by a retroviral expression system were prepared in a previous study (15). For GFP-TFEB-expressing cells, GFP-TFEB plasmids were obtained from Addgene (#38119) (53), and TFEB genes were inserted into pMRX-puromycin plasmids. Retrovirus generation was performed according to our previous study (54). HeLa (laboratory stock) cells were maintained in Dulbecco’s modified Eagle’s medium (DMEM; D6429, Sigma) supplemented with 10% FBS. Cells were cultured at 37°C in 5% CO_2._ Recombinant human VEGF165 (293-VE, R&D Systems) was used for cell culture treatment. Axitinib (S1005, Selleckchem) was added to cells at 10 μM at the same time as VEGF to block the binding of VEGF to VEGFR. Cells were treated with dibutyryl cAMP (D0627, Sigma) at 100 μM at the same time as the addition of VEGF, to mimic VEGF downstream signaling transduction. Xestospongin C (244-00721, Wako) was added along with antibiotic treatment after bacterial inoculation.

### Bacterial culture.

Streptococcus pyogenes strain NZ131 (serotype M49) was a gift from Dr. D.R. Martin (New Zealand Communicable Disease Center, Porirua, New Zealand). The SpeB-deleted NZ131 strain (SW574) was generated in a previous study (55) and maintained under 100 μg/mL spectinomycin (191-11533, Wako) selection. The A20 (serotype M1) strain was a stock in Dr. Jiunn-Jong Wu’s laboratory. This GAS strain was isolated from a blood specimen of a patient with necrotizing fasciitis (56). GAS was grown overnight at 37°C in 3 mL tryptic soy broth with 0.5% yeast extract (TSBY), then the culture was refreshed by transfer to new broth at 50-fold dilution for 3-h incubation. GAS was collected by centrifugation and resuspended in PBS, followed by cell concentration measurement with an optical density of 600 nm, with 0.4 being equal to 1 × 10^8^ CFU/mL, which was confirmed by plating. This procedure for bacterial preparation was used for all GAS infection experiments in this study. For mouse infection, refreshed culture was incubated for 6 h and then adjusted by saline for air pouch or tail vein injection.

### Bacterial infection protocol.

Cells were seeded in 24-well (4 × 10^4^ cells) or 6-well (2 × 10^5^ cells) plates and incubated with or without VEGF, axitinib, or cAMP for 2 d. The prepared bacteria were directly added to the wells at a multiplicity of infection (MOI) of 5 for endothelial cells and 25 for epithelial cells, due to the 5-fold higher internalization efficacy in endothelial cells relative to epithelial cells (14). The cell/bacteria mixture in plates was centrifuged at 500 *g* for 5 min to ensure simultaneous attachment of GAS to cells and was incubated for 30 min. After incubation, the cell culture was washed twice with PBS to remove the extracellular bacteria, and then fresh medium containing 100 μg/mL gentamicin (08975-81, Nacalai Tesque) was added to kill the remaining extracellular bacteria. Depending on the different experimental requirements, VEGF, axitinib, and/or cAMP was added again with antibiotic treatment for longer stimulation. After various time periods, cells were collected for individual experiments.

### Colony formation assay.

Cells in 24-well plates were infected with bacteria as described in the infection protocol. At 1-h and 6-h postinfection, bacteria-infected cells were washed twice with PBS and lysed by 1 mL sterile H_2_O per well. After serial dilution with PBS, the PBS containing bacteria was plated on TSBY agar plates. Colonies were counted after incubation for 24 h at 37°C. The bacterial number at 1-h postinfection was defined as the number of internalized GAS. The fold increase in GAS growth was calculated as the number of bacteria recovered at 6-h postinfection normalized against the number of internalized bacteria.

### Immunofluorescence staining.

Cells seeded at 4 × 10^4^ per well in 24-well plates with cover glasses were cultured for 2 d with or without VEGF treatment and infected with GAS according to the bacterial infection protocol. Cover glasses were coated in advance with cellular matrix (Cellmatrix type I-C, 100 μg/mL, 37°C, 30 min). At various time points postinfection, the cells were fixed with 4% paraformaldehyde (PFA), permeabilized with 50 μg/mL digitonin, blocked with 0.2% gelatin in PBS, and stained with anti-Gal-3 (M3/38, Santa Cruz), anti-LC3 (PM036, MBL), anti-LAMP-1 (H4A3, Santa Cruz), anti-FIP200 (17250-1-AP, Proteintech), or anti-Rab7 (EPR7589, Abcam) antibodies, followed by staining with secondary antibodies conjugated with Alexa Fluor 488, 568, or 647. LysoTracker (Red DND-99, Invitrogen) at 100 nM was used to stain cells for 30 min before fixation. Hoechst (33342, #639, ImmunoChemistry Technologies) at 1 μg/mL was used for cell nucleus and bacterial DNA staining. Images were obtained using a confocal microscope (TCS SP8, Leica).

### siRNA knockdown.

Universal negative control siRNA was purchased from Cosmo Bio Co. (SN-1001, BIN), and TFEB siRNA (sense 5′-CGCAUCAAGGAGUUGGGAAdTdT-3′ and anti-sense 5′-UUCCCAACUCCUUGAUGCGdTdT-3′) was purchased from Sigma-Aldrich. The transfection conditions were the same as those in our previous study (15).

### RT-PCR.

Cell pellets were collected from three wells of 6-well plates after a 2-d treatment with or without VEGF treatment, or collected after 24 h of further infection with GAS according to the bacterial infection protocol. Total mRNA extraction and quantitative PCR targeting *ATPV6*, *LAMP-1*, and *GAPDH* were performed according to our previous study (57).

### Cytosolic calcium detection by Fluo-8.

Cells were seeded on glass-bottom dishes and stimulated with VEGF or cAMP for 2 d. Cells were then stained with Fluo-8 at 4 μM in HHBS buffer (Hanks’ balanced salt solution [H9394, Sigma] containing 20 mM HEPES) for 1 h. After washout of Fluo-8 by HHBS buffer, cells were treated with VEGF or cAMP in M200 medium for another 1 h. Images were taken without fixation at 1-h poststimulation. Fluorescence intensity inside the encircled region of interest in the cytoplasmic region was measured using ImageJ.

### Western blot analysis.

The following primary antibodies were used for Western blot analysis: anti-LAMP-1 and anti-ULK1 antibodies (H4A3 and C1918, Santa Cruz); anti-Rab7 antibody (EPR7589, Abcam); anti-TFEB, anti-P-ULK1(S757), anti-P-p70-S6 kinase, anti-p70-S6 kinase, and anti-4E-BP1 antibodies (4240S, 6888S, 9234S, 2708S and 9452S; Cell Signaling); and anti-tubulin (T9026, Sigma) antibody.

### Hemolytic assay.

A hemolytic activity assay was performed using a previously established protocol (58). In brief, after refresh of the overnight-cultured medium, GAS culture supernatant was collected at 1-h postincubation for SLO or at 6-h postinfection for SLS activity analysis, because both proteins are highly expressed during these stages of GAS growth. Mouse erythrocytes were isolated from 10-week-old wild-type C57BL6/J mice purchased from Japan SLC, and prepared in 2% (vol/vol) erythrocytes in PBS for this assay. VEGF at the indicated concentrations was added to GAS culture supernatant, incubated for 30 min, and then mixed with 2% prepared erythrocytes. The hemolytic units corresponded to the supernatant of 1% erythrocytes lysed by water.

### Correlative light electron microscopy.

Cells stably expressing GFP-LC3 and mStrawberry-Gal-3 were generated in our previous study (15). Cells were cultured with or without VEGF for 2 d on glass-bottom dishes with a grid pattern (P35G-2-14-C-GRID; MatTek) and infected for 1 h with GAS at an MOI of 5 without washout. No centrifugation was performed due to the absence of a suitable rotor for this type of dish. Cells were fixed with 4% formaldehyde (FA) in PB buffer (0.1 M phosphate buffer with 2% sucrose, pH 7.4) for 30 min and 1 μg/mL Hoechst stain for 1 h at room temperature, washed with PB buffer, and observed using a confocal microscope (TCS SP8, Leica). The locations of the target cells were marked, and the same specimens were then further prepared for electron microscope ultrathin-sectioning and observation under a JEM-1400plus transmission electron microscope (JEOL).

### Conventional electron microscopy.

Cells were seeded on a cellular-matrix-coated plastic cell disc (MS-0113K, Sumitomo Bakelite) in 24-well plates and cultured with or without VEGF for 2 d. GAS infection was performed according to the bacterial infection protocol. Cell dishes were fixed at 1-h postinfection with 4% FA in PB buffer for 30 min, and the medium was then changed to 2% FA in PB buffer. The EM sampling process and 70-nm ultrasection preparation were performed as in CLEM. Ultrathin sections were observed under a transmission electron microscope (H7500 TEM, Hitachi).

### Mouse infection model.

All animal experiments were conducted in accordance with animal protocols approved by the Animal Care and Use Committee of Osaka University Graduate School of Dentistry (R-01-018-0). Eight- to 10-week-old wild-type BALB/c male mice were purchased from Japan SLC and maintained with standard laboratory food and water in the Animal Center of the Dentistry Department of Osaka University. GAS was prepared for mouse infection as described above. In the air pouch infection model, 2 mL of air was injected into the subcutaneous skin of the back, followed by GAS injection (2 × 10^8^ CFU in 200 μL PBS). The air pouch extract was collected by injecting 1 mL PBS into the air pouch and withdrawing the liquid after 5-s massage. In the bacteremia model, mouse tail veins were injected with the indicated numbers of GAS organisms that had been suspended in 200 μL PBS. To detect VEGF levels, mice in both infection models were sacrificed 24-h postinfection. VEGF and sVEGFR1 levels in mouse serum and air pouch extracts were analyzed by an ELISA kit (DY493-05 for VEGF and MVR100-1 for sVEGFR1, R&D Systems). To determine survival rates, each GAS-infected mouse in the bacteremia model was monitored daily and was injected via tail vein with 2 μg VEGF (Murine VEGF165, 450-32, PeproTech) in 200 μL saline, or orally administered 1 mg axitinib (S1005, Selleckchem) in 100 μL 0.5% carboxymethylcellulose (CMC, 039-01335, Fujifilm) at the specified number of days postinfection.

### Patient sera.

GAS-infected patient sera were collected at National Cheng Kung University (NCKU) Hospital, Taiwan. Patients consented to the use of their sera for the investigation of GAS pathogenesis, and approval was obtained from the Institutional Review Board of NKCU Hospital (IRB10708, #8800-4-03-005). The consent form also specified the reuse of remaining specimens for research related to disease caused by GAS infection. This study was approved by the Institutional Ethical Review Board of Osaka University Graduate School of Dentistry (R1-E11). Specimens were separated into invasive and noninvasive disease groups based on clinical diagnoses by Dr. Ching-Chuan Liu at NCKU Hospital. Fifty-one patients (35 male and 16 female; 14 under age 20 years, and 37 aged from 40 to 90 years) were diagnosed with severe symptoms indicating bacterial invasion, such as sepsis, necrotizing fasciitis, and streptococcal toxic shock syndrome. Thirty-six patients (22 male and 14 female; 26 under age 20 years and 10 aged from 42 to 85 years) were diagnosed with symptoms indicating a lack of bacterial invasion, including cellulitis, scarlet fever, pharyngitis, tonsillitis, and bronchopneumonia. Sera were stocked at –80°C in Dr. Liu’s laboratory at the Medical College of NCKU. Patient sera were analyzed in Dr. Liu’s laboratory. VEGF concentrations in human sera were determined using an ELISA kit (DY293B-05, R&D Systems).

### Statistics.

Statistical analysis was performed using the unpaired *t* test and one-way ANOVA. The survival rate was displayed as a Kaplan-Meier survival curve and assessed by the log-rank test. *P* values <0.05 indicated significant differences (GraphPad Prism software version 5).

### Data availability.

For original data, please contact corresponding author.
