# Supplementary figures and images for: VEGF-Mediated Augmentation of Autophagic and Lysosomal Activity in Endothelial Cells Defends against Intracellular Streptococcus pyogenes
Source: mBio. 2022 Jul 5;13(4):e01233-22. doi: 10.1128/mbio.01233-22 (PMC9426552; doi:10.1128/mbio.01233-22)

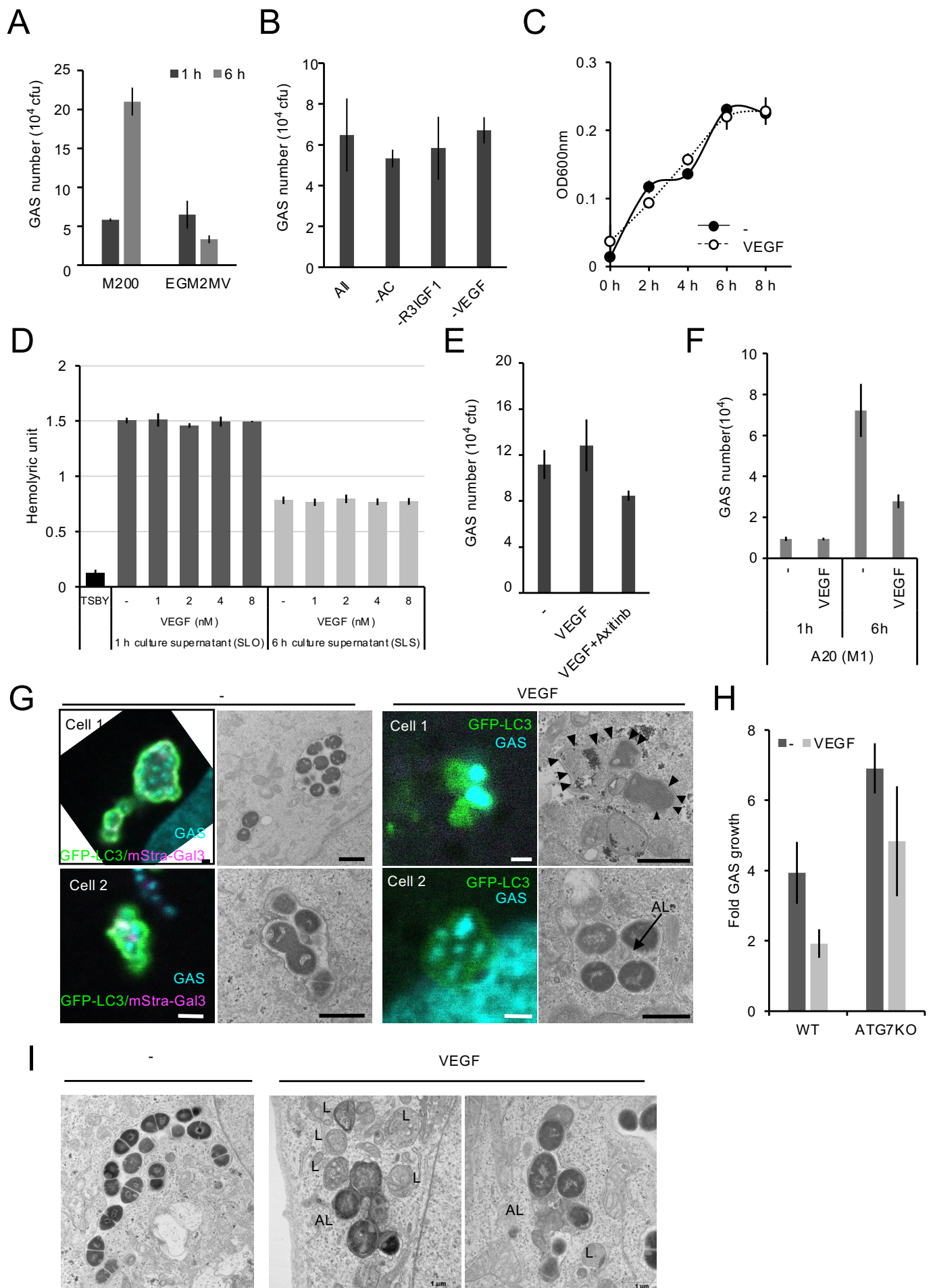

Supplementary figure 1

Supplement: FIG S1 [file mbio.01233-22-s0001.pdf]

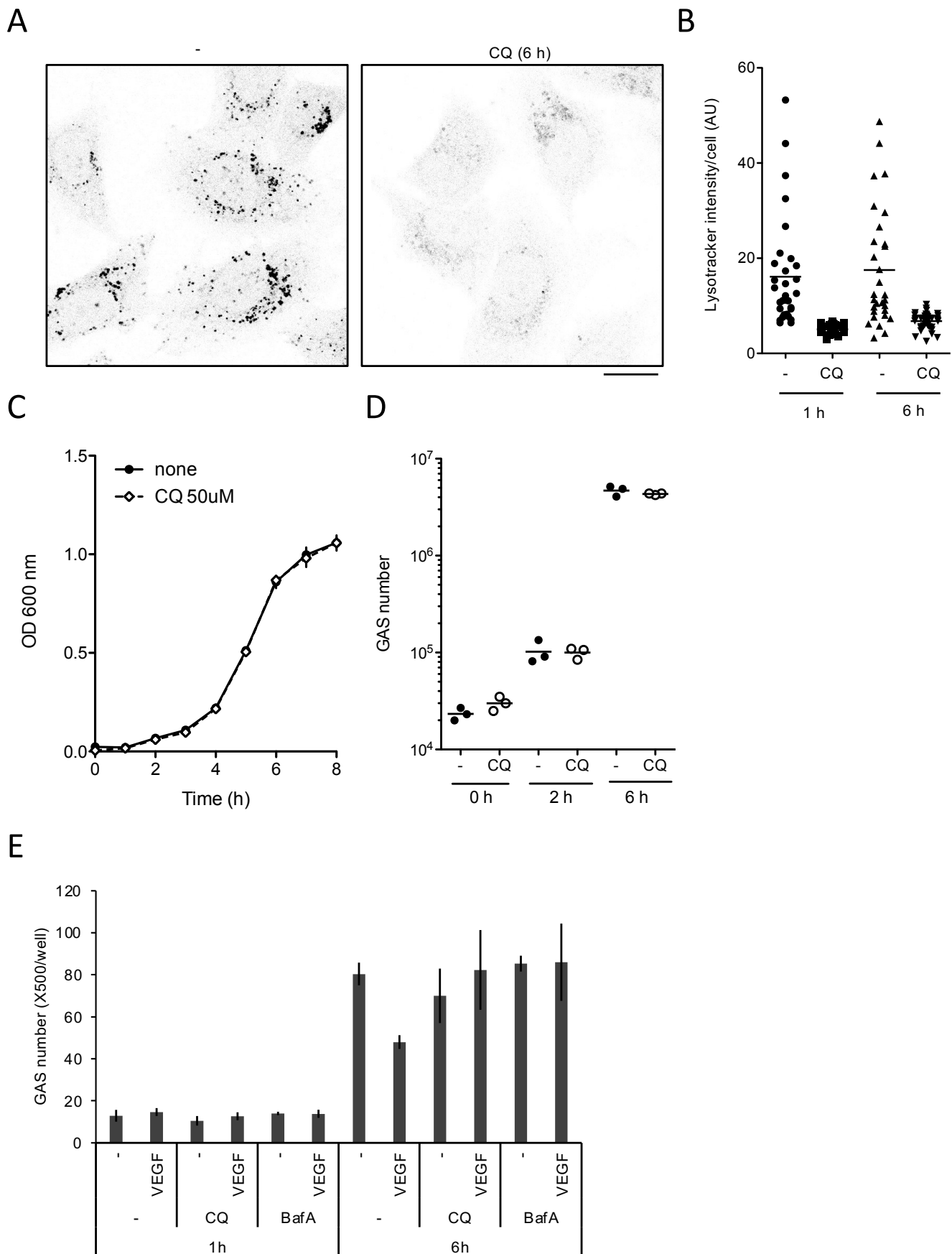

Supplement figure 2

Supplement: FIG S2 [file mbio.01233-22-s0002.pdf]

**A**

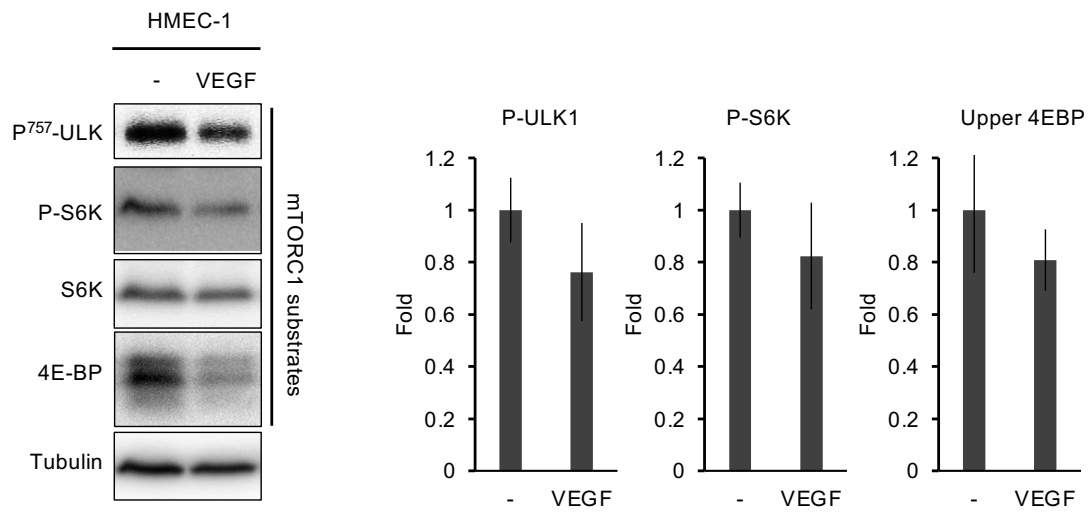

**B**

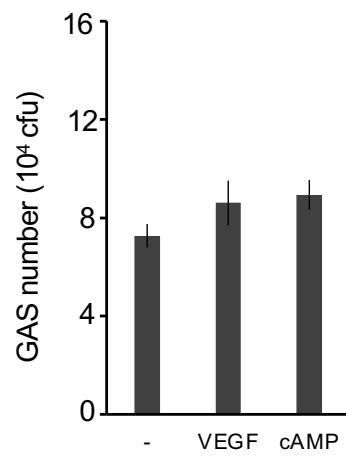

Supplementary figure 3

Supplement: FIG S3 [file mbio.01233-22-s0003.pdf]

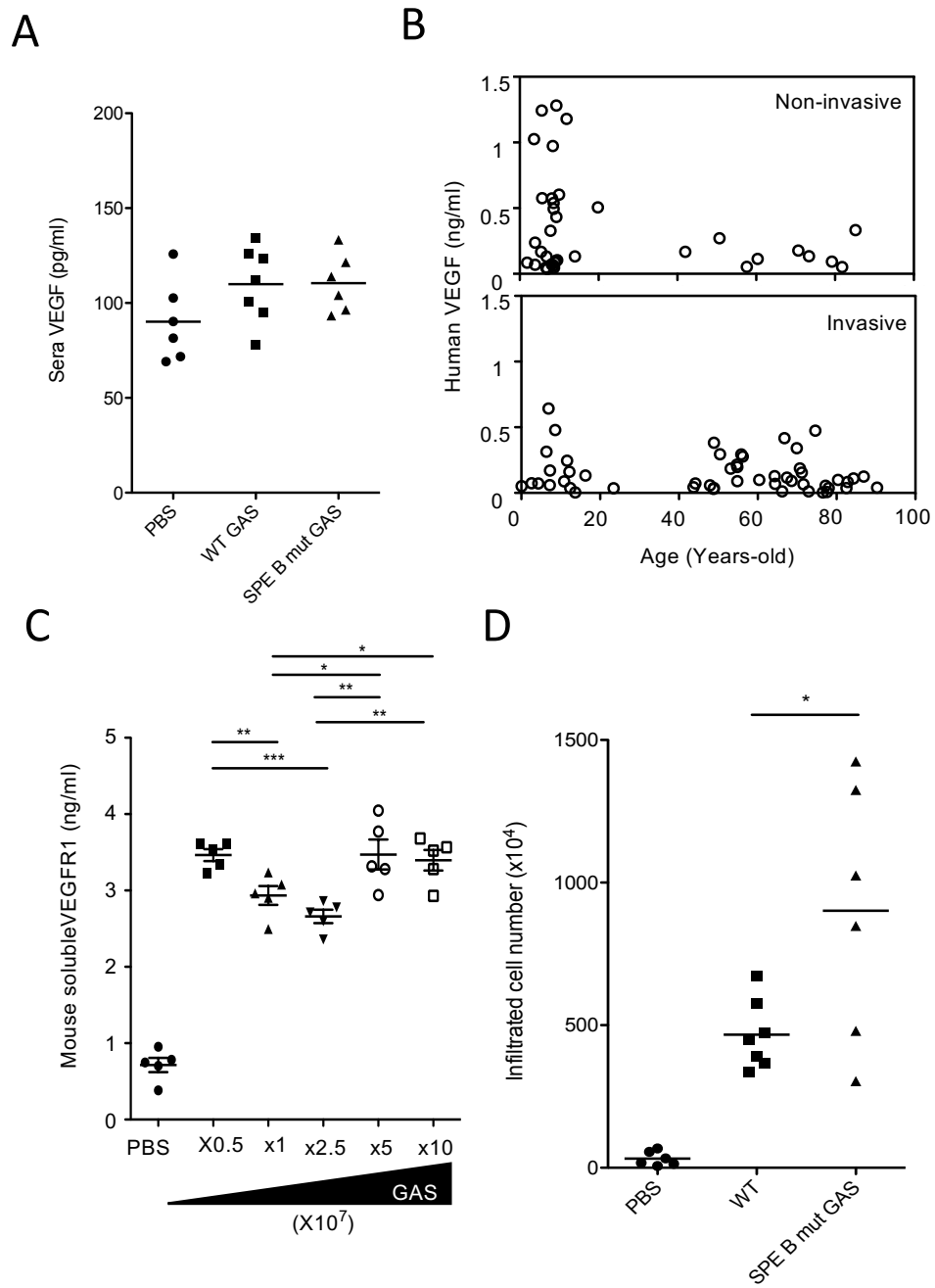

Supplementary figure 5.

Supplement: Figure S5 [file mbio.01233-22-s0005.pdf]

A

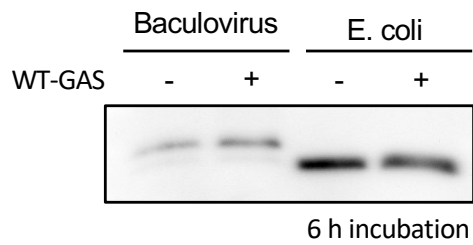

B

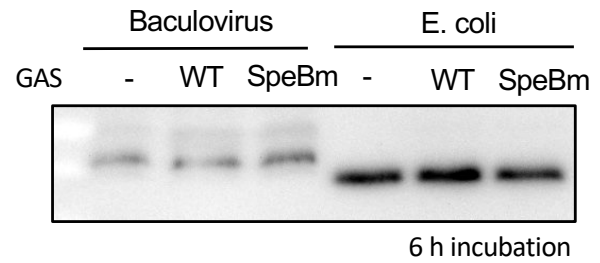

C

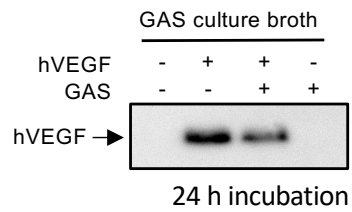

Supplementary figure 4

Supplement: FIG S4 [file mbio.01233-22-s0004.pdf]
